# Aerosol delivery through high-flow nasal therapy: Technical issues and clinical benefits

**DOI:** 10.3389/fmed.2022.1098427

**Published:** 2023-01-18

**Authors:** Cecilia Calabrese, Anna Annunziata, Domenica Francesca Mariniello, Valentino Allocca, Pasquale Imitazione, Rosa Cauteruccio, Francesca Simioli, Giuseppe Fiorentino

**Affiliations:** ^1^Department of Translational Medical Sciences, University of Campania “Luigi Vanvitelli”, Naples, Italy; ^2^Department of Intensive Care, Azienda Ospedaliera di Rilievo Nazionale dei Colli, Naples, Italy

**Keywords:** HFNC, aerosol–therapeutic, bronchodilators, high-flow nasal cannula, vibrating mesh nebulizer, jet nebulizer

## Abstract

High-flow nasal cannula (HFNC) therapy is an oxygen delivery method particularly used in patients affected by hypoxemic respiratory failure. In comparison with the conventional “low flow” oxygen delivery systems, it showed several important clinical benefits. The possibility to nebulize drugs *via* HFNC represents a desirable medical practice because it allows the administration of inhaled drugs, mostly bronchodilators, without the interruption or modification of the concomitant oxygen therapy. HFNC, by itself has shown to exert a small but significant bronchodilator effect and improves muco-ciliary clearance; thus, the nebulization of bronchodilators through the HFNC circuit may potentially increase their pharmacological activity. Several technical issues have been observed which include the type of the nebulizer that should be used, its position within the HFNC circuit, and the optimal gas flow rates to ensure an efficient drug delivery to the lungs both in “quiet” and “distressed” breathing patterns. The aim of this review has been to summarize the scientific evidence coming from “*in vitro*” studies and to discuss the results of “*in vivo*” studies performed in adult subjects, mainly affected by obstructive lung diseases. Most studies seem to indicate the vibrating mesh nebulizer as the most efficient type of nebulizer and suggest to place it preferentially upstream from the humidifier chamber. In a quite breathing patterns, the inhaled dose seems to increase with lower flow rates while in a “distressed” breathing pattern, the aerosol delivery is higher when gas flow was set below the patient’s inspiratory flow, with a plateau effect seen when the gas flow reaches approximately 50% of the inspiratory flow. Although several studies have demonstrated that the percentage of the loaded dose nebulized *via* HFNC reaching the lungs is small, the bronchodilator effect of albuterol seems not to be impaired when compared to the conventional inhaled delivery methods. This is probably attributed to its pharmacological activity. Prospective and well-designed studies in different cohort of patients are needed to standardize and demonstrate the efficacy of the procedure.

## Introduction

High-flow nasal cannula (HFNC) oxygen therapy is a well-tolerated oxygen delivery method, particularly used among patients who are hypoxemic and critically ill (1). Through a non-occlusive nasal cannula, it delivers heated and humidified air-oxygen mixtures with a variable inspired fraction of oxygen (FiO2) and at different “high flow” rates (2, 3). In comparison with the conventional “low flow” oxygen delivery systems, HFNC shows several important advantages: (1) it washes out the nasopharyngeal dead space improving the carbon dioxide clearance which is particularly useful in patients with concomitant mild hypercapnia (4, 5); (2) it induces a positive end-expiratory pressure (PEEP) that enables alveolar recruitment (6, 7); (3) it counterbalances the intrinsic PEEP (iPEEP) in patients with static and/or dynamic hyperinflation and may increase tidal volume, reduce the respiratory rate and, potentially, the work of breathing (8, 9); (4) it enables precise delivery of the FiO2 which is particularly advantageous in patients with concomitant chronic hypercapnia and/or altered respiratory drive; (5) it ensures high humidification of the inhaled gas mixtures because it favors mucus hydration and muco-ciliary clearance resulting in facilitated expectoration (10, 11).

The increased use of HFNC in numerous clinical settings has raised a new important question: is it feasible and advantageous to nebulize inhaled drugs through an HFNC circuit in comparison with the conventional inhalation drug delivery systems for patients affected by hypoxemic respiratory failure? Until now, medical doctors have adopted three different approaches to deliver inhaled medications, particularly bronchodilators, to patients undergoing HFNC oxygen therapy. One approach is to abruptly remove the patient from the HFNC circuit and then administer the inhaled drugs with either a nebulizer connected to a face-mask or mouthpiece or a pressurized metered-dose inhaler (pMDI) with a spacer. This approach could ensure enough inhaled drug is delivered but the temporary removal of the HFNC oxygen therapy might contribute to worsening the patient’s respiratory failure. Another approach is to place a mask or mouthpiece connected to the nebulizer on top of the nasal cannula but aerosol deposition was lower in comparison with the use of a mask alone due to the obstacles of the nasal cannula on the aerosol deposition (12, 13). Finally, clinicians can deliver inhaled medications by positioning a nebulizer within the HFNC circuit, thereby allowing inhaled pharmacotherapy and oxygen therapy to be performed simultaneously. In addition to all the above-mentioned producing positive results, HFNC may increase the beneficial clinical effects of the inhaled drugs, particularly of the bronchodilators nebulized through the HFNC circuit (14, 15).

However, several technical issues have been observed in regard to the nebulization of drugs *via* HFNC. The intrinsic features of HFNC may impair the delivery of inhaled drugs because the high flow gas rates could induce particle impaction both in the upper respiratory tract and in the HFNC circuit (15–18). Second, the heated and humidified air-oxygen mixtures could induce a thermodynamic effect on aerosol particles with their hygroscopic growth. The evaporation and loss in the HFNC circuit could potentially reduce the delivery of the inhaled drugs to the lungs (17, 19, 20).

**FIGURE 1:**
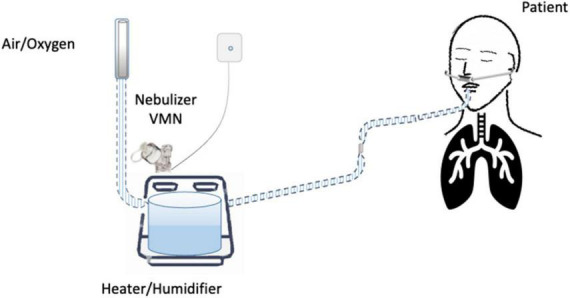
Nebulizer upstream from the heater/humidifier.

**FIGURE 2:**
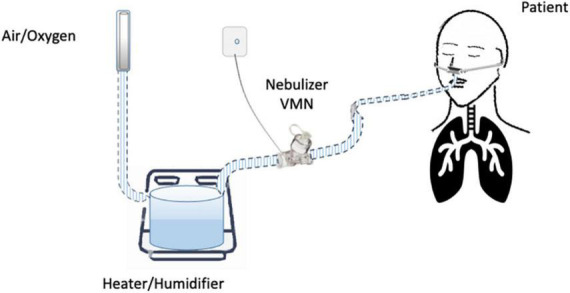
Nebulizer downstream from the heater/humidifier.

**FIGURE 3:**
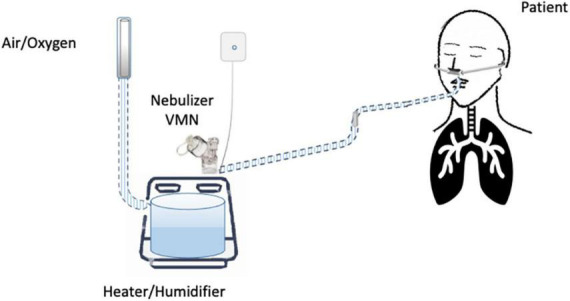
Nebulizer immediately downstream from the heater/humidifier.

Until now, the nebulization of drugs *via* HFNC has not been standardized and approved. “*In vitro*” and “*in vivo*” studies have evaluated several methodological aspects and how they can influence the pharmacological effects: (1) the type of the nebulizer, jet nebulizer (JN) or vibrating mesh nebulizer (VMN); (2) the position of the nebulizer within the HFNC circuit, close to the nasal prongs, upstream (Figure 1) or downstream (Figures 2, 3) of the humidifier; (3) the optimal gas flow rate to deliver inhaled drugs both in “quiet” and “distressed” respiratory patterns; (4) the optimal gas mixture (air/oxygen vs. oxygen/helio mixture).

On the basis of the aforementioned considerations, the aim of this article is to summarize the scientific evidence coming from “*in vitro*” studies and to discuss the results of “*in vivo*” studies regarding inhaled drug delivery *via* HFNC performed in adult subjects.

### “*In vitro*” studies

In Table 1, we summarize the most relevant benchtop studies performed in adult lung models. They have investigated the optimal setting for the nebulization including the type of interfaces, the gas flow rates in the “quiet” and “distressed” breathing patterns, and in relation to the breathing cycle, the type of nebulizer, its modifications in order to improve delivery efficiency, and the optimal position within the HFNC circuit.

**TABLE 1:** “*In vitro*” studies.

References	Study design and aim	Flow settings	Drug	HNFC device	Nebulizer	Conclusion
Perry et al. (18)	To evaluate the influences of nasal cannula size (infant, pediatric, and adult) and flow rates on the “*in vitro*” inspired dose (ID) and particle size distribution of albuterol delivered by a HFNC system.	From 3 to 40 L/min	Albuterol (2.5 mg/3 ml)	Vapotherm 2000i	Aeroneb SOLO VMN connected *via* adaptor proximal to nasal cannula and downstream from the HFNC device.	For each nasal cannula size, the ID decreased significantly with increasing the flow rates. The ID increased significantly with increasing cannula size for flow rates from 5 to 20 L/min. The amount of albuterol delivered was lower than that expected for a clinical response, with most of the albuterol dose accumulated within the adaptor.
Longest et al. (22)	To develop a device able to generate sub-micrometer aerosols with minimal depositional loss, delivered through a commercial adult nasal cannula, a divided (D) design and a divided and streamlined (DS) design of nasal cannula. The D design for the enhanced condensational growth (ECG) has been shown to improve lung delivery of nasally administered aerosols during HFNC therapy.	30 and 45 L/min	0.1% albuterol sulfate	NA	Aeroneb Lab nebulizer	The improved mixer design produced submicrometer aerosols, reduced the total deposition fraction of drug within the mixer so that the total delivery efficiency through a commercial nasal cannula reached 80–90%. The DS nasal cannula significantly improved the delivery efficiency of both submicrometer and micrometer aerosols.
Golshahi et al. (19)	To evaluate the delivery efficiency of sub-micrometer aereosol combined with condensational growth technique, using continuous inhalation flow or intermittent aerosol delivery, according to the inhalation/exhalation breathing cycles.	5, 10, 20, 40 L/min	0.2% albuterol sulfate and 0.2% sodium chloride in water	Vapotherm 2000i	Mixer and heater combined with VMN (Aeroneb SOLO)	Intermittent aerosol delivery of submicrometer condensational growth aerosols was significantly more efficient than continuous delivery.
Réminiac et al. (15)	To identify the optimal settings for the nebulization, evaluating the mass and size distribution of the aerosol emitted from nasal cannula (inhalable mass), using mesh and jet nebulizers placed at various positions within an adult HFNC circuit.	30, 45, 60 L/min	5 ml salbutamol (2 mg/ml)	Optiflow, MR850	Aeroneb SOLO VMN and two JN	Placing nebulizers within a HFNC circuit immediately upstream from the humidification chamber is the most efficient position. Higher flow rates and open mouth were associated with lower efficiency. Simulating respiratory distress did not impair drug delivery as compared to a “quiet” breathing pattern.
Dailey et al. (21)	To compare aerosol delivery using a helium-oxygen mixture (heliox) that seems to reduce the turbulence, or oxygen alone *via* HFNC at various flows in both a “quiet” and “distressed” breathing pattern.	10, 30, and 50 L/min	Albuterol sulfate (2.5 mg/3 ml)	Optiflow, MR850	Aeroneb SOLO VMN placed at the inlet of the humidifier	In a “quiet” breathing pattern, increasing flows with heliox or oxygen significantly decreased the percentages of inhaled dose of aerosol; in contrast, with a “distressed” breathing pattern, aerosol delivery was greater at 30 and 50 L/min than 10 L/min. There was a trend toward a higher inhaled dose with heliox than with oxygen, but the difference was not significant.
Bennett et al. (24)	To evaluate aerosol delivery using two nebulizer types across different drug delivery interfaces, i.e., nasal cannula, facemask, and mouthpiece connected to HFNC circuit, during simulated healthy and “distressed” breathing.	50 L/min	2-mL dose of albuterol sulfate (2 mg/mL)	Optiflow (AIRVO2)	Aeroneb SOLO VMN and a JN (Cirrus 2)	During a simulated healthy breathing and “distressed” breathing, a significantly greater aerosol dose was observed when the VMN was within HNFT connected to nasal cannula, in contrast a lower dose was delivered when either VMN or JN was within HFNT connected to a facemask or mouthpiece. Across all drug delivery interfaces, aerosol delivery was significantly greater during simulated “distressed” breathing in comparison with simulated healthy adult breathing.
Li et al. (25)	To quantify the relationship between the inhaled dose and the ratio of nasal cannula gas flow to patient inspiratory flow (GF:IF) across “quiet” and “distressed” breathing pattern.	5, 10, 20, 40, 60 L/min	Albuterol (2.5 mg/1 ml)	Optiflo, MR850	Aeroneb SOLO VMN	The inhaled dose was higher when the ratio GF:IF was <1 than with GF:IF > 1. When GF:IF ratio < 1, the inhaled dose was similar between “quiet” and “distressed” breathing.
Li et al. (26)	To compare the inhaled dose of aerosol delivered by inspiration synchronized vs. continuous generation using a VMN placed at different positions in the HFNC circuit.	5, 10, 20, 40, 60 L/min	1 ml of Albuterol (2.5 mg/mL)	Optiflow, MR850	Aeroneb SOLO VMN at the inlet of humidifier or close to patient	Inhaled dose was higher when the HFNC gas flow was set lower than 50% of patient’s inspiratory flow both in inspiration synchronized and continuous aerosol, regardless of nebulizer placement. In addition, inspiration synchronized aerosol generated 30% more inhaled dose with VMI placed at the inlet of humidifier. When HFNC gas flow was higher than 50% of patient inspiratory flow, inspiration synchronized aerosol did not add clinical advantage over continuous nebulizer placed at the inlet of humidifier.
Dutta et al. (23)	To optimize nose-to-lung aerosol delivery in an adult upper airway model considering different flow rates and synchronization parameters of aerosol generation, using a computational fluid dynamics (CFD) simulations to analyze the timing of aerosol delivery with inhalation. The study utilized the excipient enhanced growth (EEG) aerosol delivery strategy, in which relatively small particles of inhaled drugs are combined with a hygroscopic excipient to absorb humidity.	30–75 L/min	0.25% w/v albuterol sulfate (AS) and 0.25% w/v sodium chloride (NaCl)	Optiflow, MR850	Small-volume mixer-heater device	Transient CFD simulations of the small-volume mixer-heater show that the small volume aerosol bolus has a minimal spread and reaches the cannula with a time delay of only 0.2 s. Increasing the inhaled flow rate does not increase the nasal deposition even at flow rates as high as 90 L/min.

HFNC, high-flow nasal cannula; NA, not available; VMN, vibrating mesh nebulizer; JN, jet nebulizer; HFNT, high-flow nasal therapy.

In 2013, Perry et al. demonstrated that the inspired dose (ID) of albuterol decreased with smaller sized cannulas and higher gas flow rates (18). Similarly, in 2017, Dailey demonstrated that in a “quiet” breathing pattern, increasing flows significantly decreased the percentages of an inhaled dose of aerosol, while in a “distressed” breathing aerosol delivery efficiency *via* HFNC at 10 L/min was less than with the quiet breathing pattern but greater at 30 and 50 L/min. Although the mixture of helium-oxygen (heliox) determined a trend toward a higher inhaled dose in comparison with oxygen, the difference was not statistically significant (21).

In 2013, Longest developed a device to generate sub-micrometer aerosols able to improve delivery efficiency in particular when a divided and streamlined (DS) design of nasal cannula was used. Results indicate that the streamlined components can dramatically reduce depositional losses and increase an emitted dose compared with the base component system (22). In 2014, Golshahi combined sub-micrometer particles with condensational growth techniques (EEG and ECG) to reduce drug losses within the components of HFNC systems with 2 modes of intermittent aerosol delivery in comparison with continuous aerosol delivery. Significant improvements in dose delivered were observed for both condensational growth methods using intermittent aerosol delivery in comparison with continuous delivery (19).

Dutta showed that the EEG approach provides a 3-fold increase in the lung delivered dose (82.2%) compared to conventional delivery (27.5%) and the deposition in the nose-mouth-throat geometry showed only a slight increase when flow rates are higher than 90 LPM. The low volume of the new HFNC unit minimizes aerosol transit time (0.2 s) and aerosol bolus spread (0.1 s) enabling effective synchronization of aerosol generation with inhalation (23).

In 2015, Réminiac et al. demonstrated that the position of the nebulizer, both mesh and jet nebulizer, immediately upstream from the humidification chamber was the most efficient position, either in the quiet and distressed breathing pattern (15). In 2019, Bennett demonstrated the superiority in the aerosol delivery of VMN within the HFNC circuit connected to a nasal cannula in comparison with either VMN or JN connected to a facemask or mouthpiece. Regardless of the interface, aerosol delivery was significantly greater during simulated “distressed” breathing than in simulated healthy adult breathing (24).

In 2019 and 2020, Li performed two studies in which they compare the inhaled dose of aerosol generated by inspiration synchronized vs. continuous VMN *via* HFNC at different gas flows and different patient’s breathing patterns (quiet vs. distressed breathing). They demonstrated that the inhaled dose of albuterol was higher when the ratio between the gas flow and the patient inspiratory flow was <1. When the HFNC gas flow was set below 50% of the patient’s inspiratory, synchronized aerosol delivery generated 30% more inhaled dose compared to the continuous, regardless of nebulizer placement. A continuous nebulizer needs to be placed at the inlet of the humidifier. Moreover, an inhaled dose with distressed breathing was higher than quiet breathing when GF:IF was >1, while the inhaled dose was more consistent when GF:IF was <1, regardless of the patient breathing pattern (25, 26).

### “*In vivo*” studies

Most studies were performed in patients affected by obstructive lung diseases both in stable and acute clinical conditions in which the inhaled drugs nebulized *via* HFNC were short-acting bronchodilators (salbutamol and/or ipratropium bromide). Few studies have also been conducted in healthy subjects or patients affected by pulmonary hypertension and, more recently, by Coronavirus Disease 19 (COVID-19) in which inhaled epoprostenol *via* HFNC was evaluated. In Table 2, we summarize the most relevant “*in vivo*” studies.

**TABLE 2:** “*In vivo*” studies.

References	Study design and aim	Population	Flow settings	Drug	HNFC device	Nebulizer	Conclusion
Dugernier et al. (27)	To compare aerosol efficiency by using two different nebulizers, VMN or JN, through HFNC	Healthy	30 L/min	Diethylenetriaminepentaacetic acid labeled with technetium-99m (99mTc-DTPA)	Optiflow	Aeroneb SOLO VMN and JN upstream of the humidifier	The study reports poor pulmonary aerosol deposition, regardless of the type of nebulizer. However, VMN generated higher inhaled dose as compared with the JN
Alcoforado et al. (28)	To investigate the effect of gas flow and active heated humidification on the aerosol delivery efficiency *via* HFNC	Healthy	10, 30, or 50 L/min	99mTc-DTPA	Optiflow	Aerogen SOLO VMN at the inlet of the humidifier	Both flow and active heated humidity inversely impact aerosol delivery through HFNC
Bräunlich and Wirtz (29)	To compare aerosol efficiency by using conventional oral inhalation or inhalation *via* HFNC	Stable COPD	35 L/min	2.5 mg albuterol and 0.5 mg ipratropium bromide	TNI softflow 50	JN placed distally, close to the nasal prongs	Inhalation of combined bronchodilators adapted to an HFNC device is similarly effective to inhalation with a standard oral aerosol nebulizer
Reminiac et al. (30)	To investigate the bronco dilatator effect of VMN albuterol *via* HFNC compared to standard-nebulization using a JN with a facial mask and HFNC without drug	Reversible obstructive lung diseases	30 L/min	2.5 mg albuterol	Airvo™2	Aerogen Solo VMN immediately downstream of the humidifier and JN	Albuterol VMN within a HFNC circuit induces similar bronchodilation to the standard mask JN
Li et al. (31)	To determine the bronchodilator dose at which patients with stable mild-to-moderate asthma and COPD achieve similar spirometry responses as they demonstrated after bronchodilator testing using albuterol *via* MDI + spacer	COPD	15–20 L/min	Albuterol	MR 850	VMN at the inlet of the humidifier	The authors found that 1.5 mg of albuterol with VMN *via* HFNC was sufficient to induce bronchodilation and the improvement of FEV1 was similar to that obtained with MDI + spacer
Li et al. (32)	To investigate the minimally effective inhaled bronchodilator dose at various GF:IF ratios	COPD and asthma	50 L/min, GF:IF 1.0 and GF:IF 0.5	0.5, 1.0, 2.0, 4.0 mg of salbutamol	MR810	VMN, Aerogen Solo, placed at the inlet of the humidifier	A higher number of subjects responded to the doses of 0.5 mg and 1.5 mg when HFNC gas flow was set at 50% of patient peak inspiratory flow
Madney et al. (33)	To compare the pulmonary and systemic bioavailability of salbutamol delivered with different nebulizers *via* HFNC.	Stable COPD	5 L/min	5 mg Salbutamol	MR810	Aeroneb SOLO VMN or JN positioned downstream of the heated humidifier or MDI + large spacer	VMN provided higher pulmonary drug delivery than JN while no significant pharmacological benefit derived from MDI with a large spacer.
Madney et al. (34)	To compare the effects on lung function and the pulmonary and systemic bioavailability of salbutamol delivered *via* HFNC connected with 3 different interfaces (nasal cannula, face mask, and mouthpiece)	AECOPD	Titrated oxygen flow to reach sPO2 88–92%.	2.5 mg salbutamol	MR810	Aeroneb SOLO VMN positioned proximally downstream of the heated humidifier	Mouthpiece and face-mask interfaces combined with HFN provided a higher salbutamol pulmonary deposition, although with a higher systemic absorption, in comparison with nasal cannula. Despite this, the change in lung function parameters (FEV1, and FVC) post-dose inhalation was approximately similar with all the three tested interfaces and in both conditions (with or without oxygen).
Boules et al. (35)	To compare the effect of BiPAP at two difference pressure (low and high), with HFNC on pulmonary and systemic deposition of nebulized salbutamol.	AECOPD	HFNC 5 L/min or BiPAP low pressure (IPAP/EPAP 10/5 cmH2O) or BiPAP high pressure (IPAP/EPAP 20/5 cmH2O)	2.5 mg salbutamol	MR810, Fisher and Paykel or Bellavista 1000e	Aerogen Solo VMN positioned in the inspiratory limb of the BiPAP or upstream before the humidifier within the HFNC circuit	Low pressure BiPAP was more effective in the pulmonary deposition than HFNC, although the difference was not significant, while the systemic deposition, was significantly higher. High pressure BiPAP delivered the lowest amount of salbutamol.
Beuvon et al. (36)	To compare the effects on pulmonary function parameters of salbutamol nebulized *via* HFNC in comparison with that of HFNC alone	AECOPD	30 L/min	Salbutamol	NA	VMN	Salbutamol significantly improves pulmonary function parameters (FEV1, FVC, and PEF), increases hearth rate while airway resistances and breathing frequency were not significantly different
Li et al. (38)	To evaluate the impact of delivering inhaled epoprostenol (iEPO) *via* HFNC on oxygenation in spontaneously breathing subjects and in those requiring mechanical ventilation	Adult ICUs with severe hypoxemia	30–50 L/min	iEPO	MR850, Fisher and Paykel	Aeroneb^®^ Solo VMN placed upstream of a heated humidifier	Subjects’ oxygenation was improved after iEPO *via* HFNC
Ammar et al. (39)	To describe the use of iEPO administered through non-invasive ventilator routes: HFNC and NIPPV	Critically ill patients in ICU	HFNC 50 L/min or NIPPV with PS adjusted to obtain an expired tidal volume of 7–10 mL/kg of predicted body weight, whereas PEEP between 2 and 10 cm of water.	1.5 mg iEPO	MaxVenturi high-flow oxygen system and Respironics V60 Ventilator	Areogen SOLO nebulizer placed on the dry side of the humidifier for HFNC and nebulizer connected to the distal end of the circuit, closer to the ventilator for NIPPV	In critically ill patients, iEPO could be administered through a non-invasive route, improving respiratory status
Li et al. (40)	To evaluate the clinical effectiveness of iEPO and the impact of the flow titration during iEPO delivery *via* HFNC	Pulmonary hypertension and/or right ventricular dysfunction	NA	1.5 mg iEPO	MR810, Fisher and Paykel	Aeroneb^®^ Solo VMN placed at the inlet of an active heated humidifier	For patients with pulmonary hypertension and/or right ventricular dysfunction iEPO delivery through HFNC reduced mPAP and also improved oxygenation. These improvements were more evident by titrating HFNC gas flow
Kataria et al. (41)	To evaluate whether iEPO *via* HFNC prevents intubation and/or prolong time to intubation compared to controls only treated with HFNC	COVID-19 patients	≥50 L/min	iEPO	NA	VMN, placed at the humidifier	While iEPO did not significantly reduce the rate of mechanical ventilation, the time from HFNC initiation to intubation was prolonged
Li et al. (42)	A worldwide survey on the utilization of HFNC among ICU clinicians.						Post-extubation or moderate hypoxemia were major indications for HFNC. Aerosol delivery *via* HFNC was used by 24% of the participants, 30% of whom reduced flow during aerosol delivery and VMN was the most commonly used nebulizer mainly placed at the inlet of the humidifier. 40% of the participants reported placing a nebulizer with a mask or mouthpiece while pursuing HFNC whereas 33% discontinued NHF to use conventional aerosol devices.

VMN, vibrating mesh nebulizer; JN, jet nebulizer; HFNC, high-flow nasal cannula; COPD, chronic obstructive pulmonary disease; MDI, metered dose inhaler; FEV1, forced expiratory volume in the 1st second; GF:IF, the ratio of HFNC gas flow to patient peak inspiratory flow; AECOPD, acute exacerbations of COPD; FVC, forced vital capacity; BiPAP, bilevel positive airway pressure; IPAP, inspiratory positive airway pressure; EPAP, expiratory positive airway pressure; NA, not available; PEF, peak expiratory flow; NIPPV, non-invasive positive pressure ventilation; PS, pressure support; PEEP, positive end-expiratory pressure; mPAP, mean pulmonary arterial pressure; COVID-19, coronavirus disease 19; ICU, intensive care unit.

### Radiolabeled aerosol *via* HFNC in adult healthy subjects

In 2017, Dugernier et al. conducted a study on six healthy adult subjects showing the superiority of the VMN over the JN in emitting aerosol particles. They observed a poor pulmonary deposition of the radiolabeled aerosol. Within the HFNC circuit, both nebulizers were placed upstream of the humidifier. At a low flow rate of 30 L/min, <5% of the loaded dose in a VMN reached the lungs (27).

In 2019, Alcoforado et al. conducted a study aimed to compare the effect of different gas flows and active heated humidification on the deposition and distribution of radiolabeled aerosol from a VMN *via* HFNC. Twenty-three healthy subjects were randomized to receive aerosol with active heated humidification or unheated oxygen at gas flows of 10, 30, or 50 L/min. Diethylenetriaminepentaacetic acid labeled with Technetium-99m (DTPA Tc99m) was administered *via* VMN and placed at the inlet of the humidifier. Lung deposition with heated humidified gas was greater at 10 L/min than at 30 or 50 L/min. Using unheated carrier gas, the lung dose of aerosol was similar to the active heated humidification at 10 L/min, but greater at 30 and 50 L/min. Pulmonary aerosol delivery ranged from 3.5 to 17.2%. In conclusion, both gas flow rates and active heated humidity inversely impact aerosol delivery through HFNC (28).

### Bronchodilator administration *via* HNFC in adult patients affected by obstructive lung diseases

In this section, we discuss the results of seven studies conducted in patients affected by the chronic obstructive pulmonary disease (COPD) or bronchial asthma either in stable conditions or during acute exacerbations, outlining either the technical aspects of the aerosol drug delivery system *via* HFNC or the pharmacological effects obtained.

In 2018, Bräunlich and Wirtz performed the first study on 26 adult patients affected by COPD. They compared the changes in lung function parameters followed by the administration of a standard dose of albuterol and ipratropium bromide, delivered *via* a JN positioned in line with HFNC or connected with a mouth piece, as the conventional oral inhalation technique. In particular, within the HFNC (TNI soft flow 50 device medical AG, Wuerzburg, Germany), 2.5 mg albuterol and 0.5 mg ipratropium bromide were placed in a small volume JN, distallycloseto the nasal prongs, at a gas flow of 35 L/min. The authors reported a similar bronchodilator effect, as measured by post-inhalational forced vital capacity (FVC), forced expiratory volume in 1 s (FEV_1_), airway resistance (R tot), and residual volume (RV) with good patient comfort and tolerance. Positioning the nebulizer close to the nasal cannula may impair drug delivery to the patient, as it favors aerosol deposition in the cannula, resulting in a negative impact to the patient’s comfort due to aerosol nasal dripping (29).

In 2018, Reminiac et al. performed a cross-over randomized controlled trial (RCT) in 25 adult patients with stable reversible obstructive lung disease in which, on three separate days, 2.5 mg albuterol was nebulized with a JN connected to a facial aerosol mask (Standard-nebulization) or with a VMN (Aerogen Solo, Ireland) within an HFNC circuit (Airvo™2, Fisher and Paykel Healthcare) (HFNC-nebulization) and compared with a “sham” nebulization within an HFNC circuit (Control-HFNC). In particular, in the HFNC system, the VMN was positioned immediately downstream of the humidification chamber by the Airvo™Neb connector, the gas flow was set to 30 L/min, with 100% relative humidity at 37°C and medium size nasal cannula was used. Patients underwent pulmonary function tests before and after each aerosol procedure. HFNC-nebulization and Standard-nebulization displayed similar improvements in FEV_1_, functional residual capacity (FRC), and RV. Control-HFNC also determined an increase of FEV_1_ but at a lower level while no significant changes in lung volumes occurred. The authors concluded that beyond the pharmacological bronchodilation, HFNC by itself may have induced small but significant bronchodilation (30).

In 2019, Li et al. reported the dose-response relationship of albuterol delivered *via* HFNC in 42 patients with stable mild-to-moderate asthma and COPD with known positive responses to 400 mcg albuterol administered by MDI and spacer. In particular, the authors evaluated bronchodilator responses before and after escalating doubling doses of albuterol delivered by VMN, placed at the inlet of a heated humidifier, *via* an HFNC with a flow rate of 15–20 L/min. They found that 1.5 mg with VMN and HFNC was sufficient to induce bronchodilation and the improvement of FEV1 was similar to that obtained with MDI and spacer (31). The dose of albuterol sufficient to induce bronchodilatation in patients with stable COPD was lower (1.5 mg) than the 2.5 mg observed in the studies by Reminiac et al. (30) and Bräunlich and Wirtz (29). This might be explained by the utilization of a higher gas flow in their studies (30 and 35 L/min, respectively), and by the different aerosol device, which was a jet nebulizer rather than VMN in-line placement to deliver aerosol.

The ratio of HFNC gas flow to patient peak inspiratory flow (GF:IF) was found to play a key role in the trans-nasal aerosol delivery efficiency (31). In 2021, Li et al. conducted a randomized clinical trial in patients with a history of COPD or asthma and documented positive responses to inhaled bronchodilators to compare the effects of GF:IF on response to trans-nasal bronchodilator delivery. Subjects were randomized to three HFNC gas flows (50 L/min, GF:IF = 1.0, and GF:IF = 0.5) and they inhaled salbutamol at an escalating doubling dose sequence (0.5, 1.0, 2.0, and 4.0 mg diluted in a constant 2 mL volume) *via* a VMN placed at the inlet of the humidifier. They found that subjects receiving GF:IF = 0.5 responded to lower cumulative doses than subjects receiving GF:IF = 1.0 and GF = 50 L/min. In particular, in asthmatic subjects the effective dose to generate bronchodilatation responses was 1.5 mg for all three flows, while in patients with COPD, the effective dose was 1.5 mg for the group of GF:IF = 0.5, while 3.5 mg for groups of GF:IF = 1.0 and GF = 50 L/min (32). In the following studies, the authors evaluated the pulmonary and systemic deposition of the nebulized salbutamol measuring the amount of the drug in urine samples collected at 30 min and cumulatively 24 h post-inhalation, respectively. A higher amount of pulmonary deposition is suggestive of better efficacy of the drug while a higher systemic deposition is associated with more side effects.

In 2019, Madney et al. conducted a crossover RCT study in 12 patients with stable COPD aimed to compare both the relative pulmonary and systemic bioavailability of 5 mg salbutamol nebulized by either JN or VMN connected by their standard T-piece or spacer to an HFNC circuit, using low oxygen flow (5 L/min). The nebulizer with T-piece or spacer apparatus was positioned downstream of the heated humidifier. The authors observed that the VMN connected with both the T-piece or the large spacer provided higher pulmonary drug delivery than that obtained with the traditionally used JN while no significant pharmacological benefit was derived from the use of a large spacer combined with pressurized metered dose inhaler (pMDI). Similarly, systemic deposition for the JN was significantly lower than that for the VMN with T-piece only or with the large spacer (33).

In 2020, Madney et al. conducted a prospective, randomized, open-label pilot trial in 45 patients hospitalized for an acute COPD exacerbation, aimed to compare the effects of three different interfaces (nasal cannula, mouthpiece, and facemask) connected to an HFNC circuit on the pulmonary and systemic deposition of 2.5 mg of salbutamol delivered with a VMN (Aerogon Solo nebulizer, Ireland) placed proximally to the heated humidifier of an HFNC (MR810, Fisher and Paykel Healthcare, Auckland, New Zealand). Two conditions were tested with each interface on different days: with titrated oxygen flow to reach oxygen saturation between 88 and 92% or without any gas flow. Lung function measurements were performed pre- and 30 min post-bronchodilator inhalation. COPD patients showed the highest salbutamol pulmonary deposition with the mouthpiece and face-mask interfaces combined with HFN while the lowest pulmonary deposition was observed with the nasal cannula probably due to the greatest filtration capacity of the nasal route. However, mouthpiece and face-mask interfaces were associated with higher systemic absorption of the drug than the nasal cannula, potentially exposing patients to major systemic side effects. Despite this, the change in lung function parameters (FEV1 and FVC) post-dose inhalation was approximately similar with all the three tested interfaces and in both conditions (with or without oxygen) demonstrating the ability of the low dose of salbutamol delivered by the HFNC to saturate their target β2 receptors (34). In addition, the nasal cannula was the most comfortable interface. In 2022, Boules et al. conducted a study in 36 exacerbated COPD patients comparing the effect of BiPhasic Positive Airway Pressure (BiPAP) mode at two different pressure (low and high) with HFNC on the pulmonary and systemic deposition of salbutamol delivered with a VMN (Aerogen Solo) inserted in the inspiratory limb of the BiPAP or, within the HFNC circuit, upstream before the humidifier. Low pressure BiPAP delivered the highest amount of both pulmonary and systemic salbutamol, followed by HFNC and then by high pressure BiPAP. In comparison with HFNC, low pressure BiPAP was more effective in the pulmonary deposition, although the difference was not significant, while the systemic deposition, and thus the risk of side effects, was significantly higher with low-pressure BIPAP. Considering the different pulmonary drug deposition, the authors suggest that dose adjustment guidelines should be developed and used when changing from one technique to another (35).

In 2022, Beuvon et al. performed a physiological crossover study on 15 subjects with severe exacerbation of COPD admitted to an Intensive Care Unit (ICU) comparing the effects of salbutamol delivered by a VMN within an HNFC, at 30 L/min to those of HNFC alone. Salbutamol significantly improves pulmonary function parameters (FEV1, FVC, and PEF) and increased heart rate while airway resistances and breathing frequency were not significantly different (36).

### Eposprostenol administration *via* HFNC in adult patients with pulmonary hypertension or COVID-19

The high-flow nasal cannula was also used to deliver epoprostenol, a selective pulmonary vasodilator, used in clinical practice for the treatment of refractory hypoxemia associated with acute respiratory distress syndrome (ARDS), severe pulmonary hypertension (PH), and acute right-ventricular failure after cardiac surgery (37). In two retrospective studies, patients who are critically ill received iEPO *via* HFNC. Patients had improvement in their respiratory status, as measured by a mean decrease in the fraction of inspired oxygen (FiO2) and a mean increase in partial pressure of arterial oxygen to FiO2 (PaO2/FiO2) ratio. However, HFNC by itself can improve oxygenation, thus, it is difficult to isolate the benefit of iEPO from that of HFNC (38, 39).

Li et al. investigated the clinical effectiveness of iEPO delivery *via* HFNC but they also reported the clinical impact of flow titration on trans-nasal pulmonary aerosol delivery. They found that iEPO delivery *via* HFNC reduced mean pulmonary arterial pressure (mPAP) in patients with pulmonary hypertension or right ventricular dysfunction and also improved oxygenation in patients with concomitant refractory hypoxemia. These improvements were more evident among patients whose gas flow administered by HFNC was titrated during iEPO initiation than those receiving constant flow (40).

In 2022, Kataria et al. demonstrated that the aerosolization of epoprostenol with VMN in patients with COVID-19 treated with HFNC is not associated with a reduction in the rate of mechanical ventilation, although may prolong the time to invasive mechanical ventilation (41).

### The survey among ICU clinicians

In 2021, Li et al. conducted the first worldwide survey among ICU clinicians and demonstrated that only one-fourth of the medical doctors performed aerosol *via* HFNC using mostly VMN positioned at the inlet of the humidifier, more than half of the participants placed a mask or mouthpiece connected to a nebulizer on top of the nasal cannula, whereas the remaining discontinued HFNC to deliver conventional aerosol therapy. In the survey, only 30% of the participants reported decreasing the flow rate during aerosol delivery *via* HFNC due to the demonstrated inverted relationship between the inhaled dose and HFNC gas flow (42).

## Discussion

Combining aerosol delivery with HFNC oxygen therapy is a desirable medical practice for both patients and healthcare personnel, as it allows the administration of inhaled drugs, without interrupting or modifying oxygen therapy. It has been mainly utilized to deliver bronchodilators in patients with COPD affected by hypoxemic respiratory failure with or without mild hypercapnia. Although several studies have investigated the optimal setup, the nebulization of drugs *via* HFNC has not been standardized yet.

First, different types of nebulizers have been evaluated. The emitted dose and lung deposition seem to be higher with VMN than with JN (12, 33). In addition, while VMN is driven by electricity, JN requires an additional gas to operate that could alter the gas flow, modify the oxygen content, and interfere with the humidity and temperature of the HFNC system. VMN was also demonstrated to leave a lower residual volume (29). In line with that previously stated, most “*in vitro*” and “*in vivo*” studies have utilized the commercially available VMN (Aeroneb SOLO). “*In vitro*” studies have modified the VMN by combing it with a heater and mixer in order to obtain sub-micrometer aerosols, which seems to increase the total delivery efficiency in comparison with the conventional micrometer-sized aerosols. These methods have to be tested *in vivo* (19). The use of pMDIs with or without a spacer for delivering bronchodilator medications to patients undergoing HFNC represents an alternative method not yet well-evaluated. A bench study conducted by Szychowiak et al. showed that a drug delivery sufficient to induce bronchodilation can be achieved using a spacer placed close to the nasal cannula, a low flow rate, with the activation of the pMDI at the beginning of inspiration (43).

Several controversies regard the placement of the nebulizer within the HFNC circuit. Most studies suggest the upstream position of the nebulizer, before the humidifier (16, 17, 44), as preferentially adopted by ICU clinicians (42). In contrast, the placement of aerosol devices between the humidifier and the patient results in a greater aerosol deposition in the tube that can occlude the nasal prongs (29).

Most studies have shown that the percentage of the inhaled dose of the drug reaching the lung *via* HFNC is low. In the case of albuterol, its bronchodilator effect seems to be preserved, as it has been shown to improve both flows and lung volumes similarly to the conventional nebulization technique (29). Probably, the low lung deposition of albuterol is sufficient to exert its pharmacological activity; however, this could not be true for drugs whose therapeutic effect is concentration-dependent, such as antibiotics. It has to be outlined that HFNC by itself could induce a small but significant bronchodilator effect, probably due to the mechanical dilatation consequent to the increased airway pressure (30).

Another technical issue regards the optimal gas flow rate to adopt. “*In vitro*” and “*in vivo*” studies have shown that in a “quiet” breathing pattern the degree of aerosol delivery is higher with a lower flow rate (15, 25, 28, 29), probably related to the decreased turbulence and particle impaction within the HFNC circuit and airways. However, by lowering the flow rate, some physiological benefits of HFNC, such as nasopharyngeal dead space washout and decreased work of breathing, could be diminished or lost (10). In addition, the increased contact time of aerosol particles with the water vapor in the conducting circuit may further reduce the inhaled dose. In contrast to “quiet” breathing, delivery efficiency with “distressed” breathing is greater (15, 25). An “*in vitro*” study has shown that in a “distressed” breathing pattern the aerosol delivery is higher when the gas flow was set below the patient’s inspiratory flow, with a plateau effect seen at the gas flow of approximately 50% of the inspiratory flow (25). However, this measurement during HFNC is not feasible yet.

In addition, it has not been established whether an intermittent, synchronized with the breathing cycle, inhalation flow is better than a continuous aerosol in the delivery of the inhaled drug *via* HFNC. Li et al. found that, when VMN was placed at the inlet of the humidifier, inspiration synchronized aerosol generated a higher inhaled dose with HFNC gas flow set below 50% of the patient’s inspiratory flow (26).

Heliox has been described as a gas mixture able to improve the response to bronchodilators, providing a more laminar flow that reduces the impactive losses of the nebulized drug. Aerosol delivery with heliox through HFNC is associated with a higher inhaled dose at mid and high flows than oxygen, but the difference is not statistically significant; however, heliox adds cost to therapy and it should be considered mainly to reduce the work of breathing or to improve the distribution of ventilation (21).

The use of active heated humidity with HFNC is associated with lower aerosol delivery efficiency attributed to hygroscopic particle growth (45), with subsequent greater impacting losses in the circuit components and airways. At low flow (10 L/min), the pulmonary deposition of the aerosol is not influenced by the heated humidity, while at higher flows, the aerosol delivery is reduced with active heated humidity (17).

Previous “*in vitro*” studies have demonstrated that the delivered dose of the drug to the lung is directly proportional to the cannula size (17, 18). In addition, redesigned nasal cannula could improve lung delivery from nasal administrated aerosol during HFNC therapy (22).

## Conclusion

In conclusion, considering the potential benefits of aerosol delivery *via* HFNC and the technical issues, prospective and well-designed studies in a different cohort of the patient are needed to standardize and demonstrate the efficacy of the procedure.

## Author contributions

CC, GF, and AA contributed to the conceptualization. PI, RC, VA, and FS were involved in the methodology. CC and DM drafted the manuscript. GF and AA critically revised the manuscript for important intellectual content. DM and VA realized the tables and figures. All authors have read and approved the final version of the manuscript.
